# Time Course of Leptin in Patients with Anorexia Nervosa during Inpatient Treatment: Longitudinal Relationships to BMI and Psychological Factors

**DOI:** 10.1371/journal.pone.0166843

**Published:** 2016-12-28

**Authors:** Esther Stroe-Kunold, Magdalena Buckert, Hans-Christoph Friederich, Daniela Wesche, Stefan Kopf, Wolfgang Herzog, Beate Wild

**Affiliations:** 1 Department of General Internal Medicine and Psychosomatics, Medical University Hospital, Heidelberg, Germany; 2 Department of Psychosomatic Medicine and Psychotherapy, LVR-Clinics, University Düsseldorf, Düsseldorf, Germany; 3 Department of Endocrinology and Clinical Chemistry, Medical University Hospital, Heidelberg, Germany; Charité-Universitätsmedizin Berlin, Campus Benjamin Franklin, GERMANY

## Abstract

**Background:**

Leptin, a hormone secreted by adipose tissue, appears to play a major role in the homeostasis of body weight and psychobiological processes associated with anorexia nervosa (AN). However, there is scarce data on its exact influence on this disorder, in particular data over time.

**Objective:**

The present study addresses whether leptin changes during inpatient treatment play a role for treatment outcome and psychological factors in underweight AN patients.

**Methods:**

In order to understand whether leptin’s role differs in relation to AN severity, data were assessed from 11 patients with a very low BMI and a higher chronicity (high severity group; HSS; mean BMI at the beginning of the study = 13.6; mean duration of illness = 5.1 years) vs. nine with less severe symptoms (LSS; mean BMI = 16.2; mean duration of illness = 3.7 years). During the course of treatment, serum leptin concentrations were assessed weekly while weight (BMI) was assessed twice per week. Concomitantly, psychological variables were obtained by means of electronic diaries. Unconditional linear growth models were calculated to evaluate the temporal course of leptin in relation to BMI. For HSS patients, two phases of treatment (BMI < 16 and BMI ≥ 16 kg/m^2^) were investigated.

**Results:**

Leptin increased significantly with BMI in both groups of patients. For HSS patients, the increase of leptin in the first treatment phase did not predict later increases in BMI. Furthermore, the relationship of leptin and psychological factors was modulated by symptom severity. In HSS patients, higher leptin levels were associated with greater feelings of depression, anxiety, and stress whereas in LSS patients a higher leptin level showed the trend to be associated with lower psychological symptom burden.

**Conclusions:**

Our results suggest that leptin changes are differently associated with weight gain and psychological symptoms depending on the severity of starvation.

## Introduction

Anorexia nervosa (AN) is an eating disorder characterized by excessive dieting and concomitant extreme weight loss; it also includes a distorted perception of one’s own body and a distinctive fear of gaining weight. In AN, endocrine disturbances such as hyperactivity of the hypothalamus-pituitary-adrenal (HPA) axis and hypoactivity of the hypothalamus-pituitary-gonadal axis are well-known in AN. Besides alterations of central hormone systems, changes in peripheral peptides increasingly attract notice. In regard to this, peptides involved in the regulation of food intake and energy homeostasis are of particular interest.

Leptin is secreted by adipocytes and serves as a long-term satiety signal that indicates the amount of fat stored in adipose tissue. It exerts anorexigenic effects by modulating several hypothalamic neuropeptides [1]. Specifically, leptin inhibits the liberation of pro-opiomelanocortin and stimulates the release of agouti-related protein and neuropeptide Y within the arcuate nucleus. These neuropeptides exert effects on other hypothalamic areas such as the lateral hypothalamus and the paraventricular nucleus which in turn project to the nucleus of the solitary tract. Apart from this indirect path, leptin also directly reaches the nucleus of the solitary tract to amplify the short-term satiety signals from the gastrointestinal tract that are processed there [1]. In addition to its crucial role in the regulation of food intake, leptin is also involved in other physiological functions such as hematopoiesis and bone metabolism, as well as neuronal function and cognition [2, 3]. Furthermore, recent research has underlined the concept that leptin has multiple regulatory roles in the immune system [4–7].

Together with the low body weight, lower serum levels of leptin have repeatedly been found in AN patients when compared to controls (reviewed in [8, 9,10]). Several longitudinal studies assessed leptin levels during refeeding or weight recovery. The majority of these studies report an increase in leptin levels as body weight rises [11, 12, 13]. However, in two studies with more frequent (i.e., biweekly) measurements of leptin levels in adolescents with AN it was observed that during weight regain leptin reached peak levels higher than the reference range [14, 15]. It is still a matter of debate if such relative hyperleptinemia is a risk factor for problems in reaching or maintaining the target weight [8, 16–18].

In conclusion, two recent reviews have summarized that the role of leptin during weight recovery in AN should be explored further [9, 19]. Therefore, the main aim of the present study is to clarify the role and associations of leptin in patients with AN. Specifically, we have investigated the association between increases in leptin and BMI stratified by symptom severity as well as the relationship between changes in leptin and psychological symptoms during weight recovery in inpatient treatment. A basic assumption of our approach is that two important prerequisites need to be fulfilled to achieve a clarification of the role of leptin during inpatient treatment: (1) Leptin measurements should be taken repeatedly over time; (2) the association between leptin and further variables should be separately investigated for patients with a very low BMI and a high symptom severity (HSS) and patients with a lower symptom severity (LSS). This distinction is important as recent studies have shown that in AN patients with HSS endocrine and emotional responses are different when compared to patients with LSS [20, 21].

Regarding psychological symptoms of AN patients, it has been hypothesized that leptin may be associated with the psychopathology of this disorder [22]. The general so-called leptin hypothesis of depression states that leptin insufficiency and leptin resistance both may contribute to the vulnerability for depression, and that leptin thus may represent the biological link between obesity and mood disorders [23]. In the context of AN, the relationship of leptin and depressive symptoms has rarely been investigated. Based on the clinical observation that during refeeding body weight (and thus leptin) increases while simultaneously mood ameliorates Hebebrand et al. [24] hypothesized that leptin may mediate mood improvements in AN patients. To our knowledge, there is only one study that empirically investigated changes in leptin and depressive symptoms concomitantly. In contrast to the proposal of Hebebrand et al. [24], Rybakowski et al. [22] observed an inverse relationship of the rise in leptin and the amelioration of depressive symptoms in a longitudinal study in AN patients during weight recovery. However, in this study mood and leptin were assessed only twice–once at admission and again at discharge. Thus, the more frequent assessment of leptin levels and depressive mood during weight recovery may help to resolve this controversy.

### Objectives of the present study

The current study served two major aims–(1) to elucidate additional details about the course of leptin during inpatient treatment and weight recovery by differentiating between patients with HSS and LSS; (2) to determine the relationship of leptin with depressive mood and other psychopathological features in AN patients during inpatient treatment and weight recovery. To this end, leptin levels were tracked both at closely spaced time intervals (i.e., once a week) and over a longer period of time (i.e., during the entire course of inpatient treatment). Concomitantly with hormonal measurements, electronic diaries were used to additionally assess psychological variables.

## Methods and materials

### Design

The study adopted a longitudinal design. After providing a detailed description of the study to the participants, written informed consent was obtained during the first week of inpatient stay. In total, 28 AN patients were enrolled. Please note that a subgroup of these patients were subjects in previous investigations of the cortisol awakening response in AN patients [20] and of the time series of awakening cortisol [25].

At the beginning of the study, the Structured Clinical Interview for DSM-IV (SCID) was conducted. During the course of the inpatient stay, serum leptin concentrations were assessed once per week while weight (BMI) was assessed twice per week (see Table 1 for the average number of measurements). On the same day as leptin measurements were taken, patients answered questions on a handheld computer (at 1:00 pm) to assess a retrospective evaluation of the intensity of psychological symptoms in the course of the morning. The study was approved by the medical ethics committee of the University Hospital, Heidelberg.

**Table 1:** Characteristics of the analyzed sample

		HSS	LSS	Control Group
		n = 11	n = 9	n = 25
**age (years)**	***mean (SD)***	23.4 (3.8)	23.9 (4.0)	28.3 (5.5)
	***range***	(19–30)	(18–32)	(20.6–46.6)
**duration of illness (years)**	***mean (SD)***	5.1 (5.7)	3.7 (3.2)	—
	***range***	(1–17)	(1–10)	
**subtype AN-R**	***n (%)***	10 (90.9)	7 (77.8)	—
**BMI: first measurement (kg/m**^**2**^**)**	***mean (SD)***	13.6 (1.3)	16.2 (0.9)	21.2 (1.8)
	***range***	(11.8–15.7)	(14.4–17.5)	(18.0–25.0)
**BMI: last measurement (kg/m**^**2**^**)**	***mean (SD)***	17.0 (1.7)	17.1 (0.7)	—
	***range***	(14.5–19.0)	(16.0–18.4)	
**leptin: first measurement (μg/l)**	***mean (SD)***	0.69 (0.8)	2.02 (3.0)	8.9 (5.5)
	***range***	(0.1–3.0)	(0.1–9.7)	(1.6–22.2)
**leptin: last measurement (μg/l)**	***mean (SD)***	5.4 (5.9)	3.1 (1.5)	—
	***range***	(0.8–15.4)	(0.8–5.9)	
**duration of inpatient treatment (days)**	***mean (SD)***	128.9 (63.9)	68.2 (14.8)	—
	***range***	(52–231)	(42–84)	
**duration of participation (days)**	***mean (SD)***	114.8 (59.8)	64.8 (12.7)	—
	***range***	(39–223)	(39–83)	
**number of serum measurements**	***mean (SD)***	15.7 (8.4)	9.8 (1.9)	—
	***range***	(5–31)	(6–12)	
**Mental health comorbidities**^**a**^ **(at enrolment)**	***n(%)***			
major depression (current)		4 (36.4)	6 (66.7)	0
minor depression (current)		1 (9.1)	1 (11.1)	0
panic disorder (current)		1 (9.1)	3 (33.3)	0
generalized anxiety (current)		1 (9.1)	0	0
social phobia (current)		2 (18.2)	0	0
Obsessive compulsive (current)		0	1 (11.1)	0

HSS = patient group with high symptom severity; LSS = patient group with less severe symptoms; AN-R: restrictive subtype; SD = standard deviation

^a^ diagnoses based on the Structured Clinical Interview for DSM-IV

Note: Out of N = 28 AN patients enrolled in the study, only N = 20 could finally be included in the data analysis (for reasons described in the text). Only the participants finally analyzed are described in this table.

### Participants

All participants were female AN inpatients meeting the DSM-IV criteria, over 18 years old with a BMI > 11 kg/m^2^ and sufficient physical and mental health to participate. Of 28 recruited patients, the data of 20 patients could eventually be included in the longitudinal analysis. Of the eight excluded patients, serum assessment was not possible for two of the patients for physical reasons while six patients dropped out of study participation. Subjects who passively or actively refused participation did not differ significantly from those patients included in data analysis with respect to age (mean age of the participants: 23.6 years; non-participants: 21.6 years; *p* = 0.34) or BMI at the beginning of the study (mean BMI of the participants: 14.8; non-participants: 14.1; *p* = 0.38; unpaired *t*-test, two-tailed).

The 20 included AN patients were admitted to two different wards. An essential part of the inpatient treatment was weight recovery. Patients were held responsible for gaining weight according to a self-imposed contract. For discharge, an optimal BMI of 18.5 was targeted; however, this aim was frequently not attained. Eleven patients were recruited from an integrated psychosomatic and internal medicine ward (a group with high symptom severity, HSS). This ward–specialized in the treatment of AN patients with a very low BMI and a longer illness duration–provided an intensive therapeutic schedule within a protected environment (e.g. supervised meal intake, weight management). The remaining nine patients were recruited from a psychotherapeutic ward (a group with less severe symptoms, LSS) that provided a multimodal psychodynamic-oriented treatment. According to actual BMI, the patients were held responsible to, at the very least, maintain or gain weight according to a self-imposed contract.

Admission to the two different wards was determined by the severity and chronicity of the illness. Patients with a low BMI, medically endangered patients, and patients with either a chronic course of the disorder or frequent relapses were admitted to the HSS ward. The characteristics of the analyzed sample (N = 20) are described in Table 1.

Twenty-five healthy women served as control group for the comparison of leptin levels with patients. These participants were recruited via advertisements within the context of another study. They underwent the SCID to exclude diagnoses of mental disorders. All participants were required to have a BMI between 18 kg/m^2^ and 25 kg/m^2^ and to be 18 years or older. Furthermore, participants currently dieting or experiencing significant weight fluctuations in the past 6 months were excluded. All participants provided written informed consent.

### Biochemical analyses

In AN patients, blood samples were taken in the course of the morning (between 10 and 12 a.m.). Blood samples of controls were taken at 11 a.m. after a standardized breakfast at 9:30 a.m. Once the blood was centrifuged under cold conditions, the serum was separated and stored at—80°C. Leptin was measured using a commercial kit based on a sandwich ELISA assay from Beckman Coulter (Sinsheim, Germany) with a detection limit of 0.2 ng/ml.

### Electronic diary measurements of psychological aspects

After enrolment, the patients received an electronic diary and training on how to use it [26]. Throughout the course of her inpatient treatment, the patient completed the diary on a daily basis before going to sleep. An alarm signal was used to remind her to complete the data assessment. The items were rated on a visual analogue scale (VAS) with bipolar labels. The marked points were converted by the computer program to a numeric scale, from 0 to 100, visible to the patient while completing the questionnaire. Handheld computers of type ARCHOS 5 Internet Tablet were used as electronic diaries. The “Octopus Mobile Survey Tool” was used as software for the mobile computers.

In the diary, questions assessing the degree of depression, anxiety, stress, pro-anorectic beliefs, and eating concerns were implemented. The items were adapted from psychometric questionnaires. Depression and anxiety were assessed by items taken from the Patient Health Questionnaire (PHQ-4) [27]. The intensity of pro-anorectic beliefs was obtained by an item taken from the Pros and Cons of Anorexia Nervosa scale (P-CAN) [28]. Regarding the assessment of eating concerns, an AN core symptom assumed to be particularly relevant to the course of inpatient treatment, two items taken from the Eating Disorder Examination Questionnaire (EDE-Q) [29, 30] assessed preoccupation with food and fear of losing control over eating. The particular items were chosen according to their psychometric properties. Regarding the assessment of stress the patients were asked to directly rate their stress level in the course of the morning. The diary items and details on item selection are provided in Table 2.

**Table 2:** Items assessing psychological aspects implemented in the electronic diary

	item	scale
**depression**	“Over the last few hours, I have been feeling down, depressed, or hopeless”	PHQ-4
		subscale “depression” (r_it_ = 0.71)
**anxiety**	“Over the last hours I have been feeling nervous, anxious, or on edge.”	PHQ-4
		subscale “anxiety” (r_it_ = 0.63)
**stress**	“How would you rate your stress level this morning?”	^a^
**pro-anorectic beliefs**	“Over the last few hours, my anorexia has been making me feel secure.”	P-CAN
		subscale “safe/secured” (FL = 0.78)
**preoccoupation with food**	“Over the last few hours, thinking about	EDE-Q
	food, eating or calories has been making it very difficult to concentrate on things I am interested in (for example: working, following a conversation, or reading).”	subscale “eating concern” (r_it_ = 0.77)
**fear of losing control**	“Over the last few hours, I have been experiencing a definite fear of losing control over eating	EDE-Q
		subscale “eating concern” (r_it_ = 0.77)

In representing the variable of interest (*r*_*it*_: item-to-total correlations; *FL*: factor loadings), items were chosen according to their psychometric properties. Additionally, we took into consideration which items would be clinically most adequate for daily assessment.

*PHQ-4*: Patient Health Questionnaire-4 (Löwe et al., 2010).

*P-CAN*: Pros and Cons of Anorexia Nervosa (P-CAN) scale (Serpell et al., 2004).

*EDE-Q*: Eating Disorders Examination Questionnaire (Fairburn and Beglin, 1994; Hilbert et al., 2007).

^a^ Item not taken from a questionnaire.

### Data analysis

Data analysis was conducted using SAS 9.4® (SAS Institute Inc., Cary, NC, USA). Logarithmic (log10) transformation was performed for leptin. Independent t-tests were used for group comparisons. The PROC MIXED procedure was used to fit individual growth models (i.e., multilevel models) for log10 leptin dependent on BMI increase and BMI trends over time. By calculating unconditional linear growth models, we obtained estimates of the average slope (i.e., linear time trend) for the entire sample of participants. The slope is the rate of change in a regression line. For example, when running a regression analysis with BMI as predictor and leptin as dependent variable, the slope is the mean amount of change in leptin-values when BMI increases by one unit. Thus, a positive slope would reflect an increase in leptin, while a negative slope would reflect a decrease in leptin when BMI increases. An individual growth model estimates the average intercept and slope of a regression line, including all participants. However, as each participant has repeated measurements in all variables, the model estimates an individual regression line for each participant and then calculates average parameters for the whole sample. It thus allows intercepts as well as slopes to vary across individuals [31]. Concerning the relationships between time trends in leptin and BMI as well as log10 leptin and psychological processes, Pearson correlations were calculated.

For HSS patients, analysis was conducted separately for the two phases of inpatient treatment. These phases were chosen according to the DSM-V specification of AN severity—during Phase I the severity of the patients was considered as “severe” or even “extreme” (BMI < 16), while during Phase II the severity ranged from “moderate” to “mild” (BMI ≥ 16). For LSS patients, data were analyzed for Phase II only (due to their BMI range).

## Results

### Comparison of leptin levels between patients and controls

At the start of the study, mean leptin levels of patients were significantly lower than mean levels of controls (8.9 ± 5.5 μg/L; control group versus HSS: t(34) = 9.5; p < .001; control group versus LSS: t(32) = 5.6; p < .001). This was still the case at the end of treatment (control group versus HSS: t(11.8) = 2.3; p = .043; control group versus LSS: t(32) = 3.7; p = .001). Mean BMI was higher in controls (21.2 ± 1.8 kg/m^2^) compared to both patient groups at both measurement points (all p < .001). The respective descriptive statistics for patients are provided in Table 1.

### Change in leptin during inpatient treatment

Table 3 displays linear time trends in BMI (Model 1) and the change in leptin dependent on BMI (Model 2) during inpatient treatment.

**Table 3:** Significant linear time trends of longitudinal regressions during inpatient treatment

	treatment phase	number of patients	Model 1		measurements per patient	Model 2		measurement per patient
			BMI = time		*mean (SD)*	Leptin = BMI		*mean (SD)*
			slope	p-value		slope	p-value	
HSS	I: BMI<16	11	**+0.12**	**< .0001**	19.55 (15.10)	**+0.32**	**0.0543**	9.91 (7.41)
	II: BMI≥16	9	**+0.10**	**< .0001**	13.44 (11.08)	**+1.63**	**0.0002**	7.11 (5.53)
LSS	II: BMI≥16	9	**+0.05**	**0.0038**	16.44 (5.41)	**+1.93**	**0.0036**	8.00 (2.18)

HSS = patient group with high symptom severity; LSS = patient group with less severe symptoms

Treatment phases were chosen according to the DSM-V specification of AN severity (see details in text).

Model 1 / Model 2 specify the longitudinal regression model.

Significant slopes are printed in bold, trend-level significant slopes in italic.

**Fig 1:**
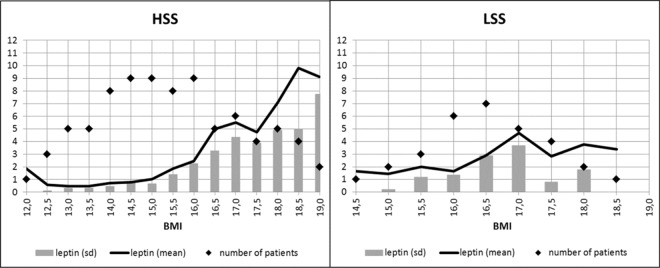
Average leptin serum concentrations (mean values) plotted against the BMI status of HSS and LSS patients (in increasing order). HSS = patient group with high symptom severity; LSS = patient group with less severe symptoms. Leptin was measured in μg/ l. Standard deviations (sd) are plotted in grey, number of measurements are plotted as black dots. Note that statistical analyses in the LSS group were performed starting with BMI = 16 kg/m^2^.

As can be seen in Table 3, all slopes were positive and highly significant, except for the slope of leptin against BMI in HSS patients during the first treatment phase which reached only trend-level significance. In HSS patients, BMI increased more strongly during the first treatment phase as compared to the second treatment phase. In line with the treatment foci of the two different wards, in LSS patients only small BMI increases were observed. Along with BMI, leptin increased in both groups during treatment, with a more pronounced increase when the BMI was ≥ 16 kg/m^2^ (see also Fig 1).

### Relationship of leptin and weight gain

For the HSS group, we also investigated whether the increase in leptin during the first treatment phase influenced weight gain during the second treatment phase. However, the partial Pearson correlations (controlled for age) between the time trend of leptin in the first treatment phase with the increase in BMI during the second treatment phase (*p* = 0.35) as well as with the total increase in BMI (*p* = 0.28) were not significant. Further, the partial Pearson correlation (controlled for age) between individual peak levels of leptin and total BMI increase during treatment was remarkably high (r = 0.86; *p* = 0.0015).

### Relationship of leptin and psychological variables during inpatient treatment

Correlations of leptin and psychological variables are provided in Table 4. As can be seen, treatment phase and severity of illness appear to have an influence on the association between leptin and psychological symptoms. During the first treatment phase leptin levels were significantly positively related to the subjective ratings of depression, anxiety, and stress in HSS patients. Similar relationships were observed for the two items measuring AN-specific eating concerns, i.e., preoccupation with food and fear of losing control over eating behavior. Interestingly, no such associations were found in the second treatment phase. In contrast, negative associations of leptin and subjective ratings of depression, anxiety, and stress were observed in LSS patients. It should be noted, however, that these correlation coefficients reached trend-level significance only. In summary, the relationship of leptin and psychological variables measuring depressed and anxious mood appears to change its direction depending on illness severity and treatment phase.

**Table 4:** Partial Pearson correlations (controlled for BMI) between log10 leptin and psychological processes during inpatient treatment

			Partial Pearson correlations (controlled for BMI) of log10 leptin with the following factors											
sample	treatment phase	number of patients	depression		anxiety		stress		pro-anorectic beliefs		eating concernpreoccupation with food		fear of losing control	
			r	p-value	R	p-value	R	p-value	R	p-value	R	p-value	R	p-value
HSS	I: BMI < 16	11	**0.25**	**0.0119**	**0.35**	**0.0004**	**0.31**	**0.0015**	0.04	0.7040	**0.32**	**0.0014**	***0*.*18***	***0*.*0675***
	II: BMI ≥ 16	9	-0.01	0.9388	0.01	0.9245	-0.03	0.8349	-0.02	0.8678	0.19	0.1360	-0.14	0.7352
LSS	II: BMI ≥ 16	9	***-0*.*24***	***0*.*0555***	***-0*.*22***	***0*.*0806***	***-0*.*22***	***0*.*0830***	-0.09	0.4748	-0.15	0.2513	-0.04	0.7352

HSS = patient group with high symptom severity; LSS = patient group with less severe symptoms; r = Pearson’s correlation coefficient

Significant slopes are printed in bold, trend-level significant slopes in italic.

## Discussion

To our knowledge, this is the first study to investigate the time course of leptin during inpatient treatment and weight recovery by means of closely-spaced serum assessments over the complete period of inpatient treatment in AN patients with different levels of symptom severity. In summary, the longitudinal analysis revealed that the increase in BMI was associated with an increase in leptin in HSS as well as LSS patients. The main finding was that there was only a weak association with a smaller slope between BMI and leptin for weight gain below a BMI of about 15–16 kg/m^2^. Above a BMI of 16 kg/m^2^ weight recovery in LSS and HSS showed the expected close and strong association between BMI and leptin.

Furthermore, in HSS patients, the increase in leptin in the first treatment phase was not associated with weight gain in the second treatment phase but the correlation between individual peak levels of leptin and total BMI increase during treatment was very high. Furthermore, our findings suggest that the relationship of leptin and psychological factors could be modulated by the severity of the illness.

As expected leptin levels at the beginning of the study were considerably lower in underweight AN patients compared to healthy controls. During treatment, the increase in BMI was associated with an increase in leptin in both patient groups. However, at the end of treatment, leptin levels were still lower in patients, probably because their BMI was also still significantly lower than controls`at this point in time. This finding is consistent with previous reports that assessed leptin less frequently [11, 12, 13, 32]. However, our longitudinal approach allows further specifications. We observed that for HSS patients the increase in leptin was more pronounced in the second treatment phase than in the first treatment phase. The first treatment phase is mainly characterized by refeeding, which is also reflected in the marked increase of BMI during this period. It may be hypothesized that the weaker increase of leptin during this phase may be due to an adaptive response to starvation [33].

Based on early observations that leptin levels can reach disproportionally high levels during weight recovery [14, 15], and that high leptin levels at discharge were predictive for later relapse [34], it has been hypothesized that a too rapid increase in leptin may inhibit further weight gain [8]. Other studies, however, found no evidence for an inhibiting effect of leptin during weight recovery [35–37]. Similarly, our data did not reveal any evidence in favour of this hypothesis. In this study, the increase in leptin in the first treatment phase was not related to later increases in BMI. Interestingly, individual peak leptin levels were strongly positively correlated with increases in BMI over the course of inpatient treatment. Here, we could tentatively infer that a higher variability in leptin levels in HSS AN patients could be associated with a more significant weight gain. However, this is a new hypothesis which should be evaluated in further studies.

Regarding the association between leptin levels and psychological self-assessment of the patients, we found that the correlation coefficients between current leptin levels and items assessing depression, anxiety, and stress were negative (trend-level significant) in LSS patients, whereas the opposite pattern was observed in HSS patients during the first treatment phase. Negative associations of leptin and depression as well as anxiety have been reported outside the context of AN. Animal studies provide strong evidence for antidepressant and anxiolytic effects of leptin [23, 38, 39]. Of note, Lawson et al. [40] found a negative association of leptin levels and depression, anxiety, and perceived stress in humans across a wide spectrum of body weight; besides normal-weight and overweight persons their sample also included AN patients with a mean BMI of 18.3 ± 0.3 kg/m^2^. Thus, the negative correlations of leptin and concomitant depressive mood, anxiety, and stress found in LSS patients who had a mean BMI of 16.2 ± 0.9 kg/m^2^ before treatment are well in line with this study. Leptin´s effects on the mesolimbic reward system, and its interactions with the serotonergic system as well as with the HPA axis (see for example the reviews by [19, 23, 41]) may underlie these negative correlations.

In contrast, in HSS patients the correlation coefficients showed the opposite direction during the first treatment phase when patients had a BMI < 16 kg/m^2^. Similarly, a positive association of leptin and AN-specific psychopathology (i.e., eating concerns) was observed in HSS patients during the first treatment phase. Interestingly, a recent study by Ruscica et al. [42] also found positive correlations of leptin and eating disorder symptoms in an AN sample with a median BMI of 13.7 kg/m^2^. The only previous pre-post study that assessed leptin and depression simultaneously in AN patients found that higher increases in leptin from the time of admission to the time of discharge were associated with less improvements in clinician-rated depression scores [22]. The AN sample of Rybakowski et al. [22] had a mean BMI of 14.45 ± 0.90 kg/m^2^ at the beginning of the study. Thus, this result fits to the positive correlation of leptin and depression ratings observed for HSS patients in our study.

Taken together, our findings of negative as well as positive associations of leptin and psychological symptoms are in line with reports in the literature. Our results present correlations only and we therefore cannot infer causative associations. However, the question remains: How can the divergent directions of correlations obtained in this study that depend on AN severity be explained? It is tempting to speculate whether or not alterations of abilities that regulate emotions could be responsible for the inverse association observed in HSS patients as compared to LSS patients. It is well known that AN patients have difficulties recognizing and regulating their emotions [43, 44]. It has been proposed that AN behaviour actually serves as an emotion regulation strategy, alleviating mood by numbing negative feelings in the short run, but leading to starvation-induced mood deteriorations in the long run, thus creating a vicious cycle [21, 45, 46]. Therefore, severely ill AN patients may be emotionally numb before starting treatment, and leptin may possibly enable these patients to recognize their (negative) emotional state. Such a mechanism would explain the observed negative correlation of leptin in relation to depression, anxiety, and stress in HSS patients during the first treatment phase. Although leptin has not yet been investigated in the context of emotion recognition, there is some indirect evidence that could suggest a potential moderating role of this adipokine in emotion processing. Leptin receptors are found at extra-hypothalamic sites such as the limbic system [38, 47], i.e., brain areas that are involved in emotion recognition [48], and leptin appears to enhance cognitive functioning and neuroplasticity [2, 49]. However, our study cannot infer whether or not leptin moderates or mediates specific associations between emotions and cognitive functions in AN patients. Clearly, more research is needed to confirm a hypothesis regarding a moderating or mediating role of leptin.

Recently, a strong claim for the initiation of clinical trials of leptin treatment in AN patients has been made [24]. Our results add a caveat to this suggestion; even if leptin may be a helpful pharmaceutical in less severely ill AN patients—and possibly also alleviate depressed mood in these patients—leptin administration could have detrimental effects on mood and anxiety in those AN patients who are severely ill and emaciated. Consequently, it seems necessary to consider initial BMI levels and symptom severity before initiating the treatment of anorexia with leptin, and to closely monitor psychic factors during such a treatment.

### Limitations

Our study has some limitations. First, only serum levels of total leptin were assessed. Nevertheless, it is possible that during treatment the density and/or sensitivity of leptin receptors may also change. It may therefore be helpful to distinguish between free and bound leptin [42] in future studies, and to include measures of leptin transmembrane receptor binding to get a more complete picture of leptin actions during AN treatment. Furthermore, our sample size could be considered rather small. On the other hand, however, the number of measurement points was very high, thereby enabling the close tracking of individual leptin levels and the reliable estimation of trends over time.

### Conclusion

In the current study serum levels of leptin and concomitant psychological variables were assessed weekly in two groups of anorectic patients across a broad BMI spectrum during inpatient treatment and weight recovery. Results underline the validity of leptin as a possible biomarker for weight gain during inpatient treatment of AN. Furthermore, our findings suggest that leptin changes are differently associated with weight gain and psychological symptoms such as depression, anxiety, and perceived stress depending on the severity of starvation. Further research is required to establish the mechanisms behind this observation.

## Supporting Information

S1 File Leptin data of both the patient and the control group. (XLSX)

S2 File Repeated measurement data including leptin, BMI, and psychosocial variables. (XLSX)

## References

[1] Morton G, Cummings D, Baskin D, Barsh G, Schwartz M. Central nervous system control of food intake and body weight. Nature. 2006;443(7109):289–95. 10.1038/nature05026 16988703

[2] Morrison CD. Leptin signaling in brain: A link between nutrition and cognition? Biochimica et Biophysica Acta (BBA)—Molecular Basis of Disease. 2009;1792(5):401–8. 19130879 10.1016/j.bbadis.2008.12.004 PMC2670357

[3] Fantuzzi G, Faggioni R. Leptin in the regulation of immunity, inflammation, and hematopoiesis. J Leukoc Biol. 2000;68(4):437–46. Epub 2000/10/19. 11037963

[4] Procaccini C, La Rocca C, Carbone F, De Rosa V, Galgani M, Matarese G. Leptin as immune mediator: Interaction between neuroendocrine and immune system. Dev Comp Immunol. 2016. Epub 2016/06/12. 10.1016/j.dci.2016.06.006 27288847

[5] Procaccini C, Pucino V, Mantzoros CS, Matarese G. Leptin in autoimmune diseases. Metabolism. 2015;64(1):92–104. Epub 2014/12/04. 10.1016/j.metabol.2014.10.014 25467840

[6] La Cava A, Matarese G. The weight of leptin in immunity. Nat Rev Immunol. 2004;4(5):371–9. Epub 2004/05/04. 10.1038/nri1350 15122202

[7] Naylor C, Petri WA Jr. Leptin Regulation of Immune Responses. Trends Mol Med. 2016;22(2):88–98. Epub 2016/01/19. 10.1016/j.molmed.2015.12.001 26776093

[8] Monteleone P, Castaldo E, Maj M. Neuroendocrine dysregulation of food intake in eating disorders. Regulatory Peptides. 2008;149(1–3):39–50. 10.1016/j.regpep.2007.10.007 18582958

[9] Tortorella A, Brambilla F, Fabrazzo M, Volpe U, Monteleone AM, Mastromo D, et al Central and Peripheral Peptides Regulating Eating Behaviour and Energy Homeostasis in Anorexia Nervosa and Bulimia Nervosa: A Literature Review. European Eating Disorders Review. 2014;22(5):307–20. 10.1002/erv.2303 24942507

[10] Modan-Moses D, Stein D, Pariente C, Yaroslavsky A, Ram A, Faigin M, et al Modulation of Adiponectin and Leptin during Refeeding of Female Anorexia Nervosa Patients. The Journal of Clinical Endocrinology & Metabolism. 2007;92(5):1843–7. 17327386 10.1210/jc.2006-1683

[11] Bosy-Westphal A, Brabant G, Haas V, Onur S, Paul T, Nutzinger D, et al Determinants of plasma adiponectin levels in patients with anorexia nervosa examined before and after weight gain. European Journal of Nutrition. 2005;44(6):355–9. 10.1007/s00394-005-0533-3 15793670

[12] Haas V, Onur S, Paul T, Nutzinger DO, Bosy-Westphal A, Hauer M, et al Leptin and body weight regulation in patients with anorexia nervosa before and during weight recovery. The American Journal of Clinical Nutrition. 2005;81(4):889–96. 15817868 10.1093/ajcn/81.4.889

[13] Holtkamp K, Mika C, Grzella I, Heer M, Pak H, Hebebrand J, et al Reproductive function during weight gain in anorexia nervosa. Leptin represents a metabolic gate to gonadotropin secretion. Journal of Neural Transmission. 2003;110(4):427–35. 10.1007/s00702-002-0800-x 12658369

[14] Ballauff A, Ziegler A, Emons G, Sturm G, Blum W, Remschmidt H, et al Serum leptin and gonadotropin levels in patients with anorexia nervosa during weight gain. Molecular Psychiatry. 1999;4(1):71–5. 10089013 10.1038/sj.mp.4000478

[15] Hebebrand J, Blum WF, Barth N, Coners H, Englaro P, Juul A, et al Leptin levels in patients with anorexia nervosa are reduced in the acute stage and elevated upon short-term weight restoration. Molecular Psychiatry. 1997;2(4):330–4. Epub 1997/07/01. 9246674 10.1038/sj.mp.4000282

[16] Södersten P, Nergårdh R, Bergh C, Zandian M, Scheurink A. Behavioral neuroendocrinology and treatment of anorexia nervosa. Frontiers in Neuroendocrinology. 2008;29(4):445–62. 10.1016/j.yfrne.2008.06.001 18602416

[17] Holtkamp K, Herpertz-Dahlmann B, Mika C, Heer M, Heussen N, Fichter M, et al Elevated Physical Activity and Low Leptin Levels Co-occur in Patients with Anorexia Nervosa. The Journal of Clinical Endocrinology & Metabolism. 2003;88(11):5169–74. 14602745 10.1210/jc.2003-030569

[18] Miller KK. Endocrine dysregulation in anorexia nervosa update. J Clin Endocrinol Metab. 2011;96(10):2939–49. Epub 2011/10/07. PubMed Central PMCID: PMCPMC3200238. 10.1210/jc.2011-1222 21976742 PMC3200238

[19] Monteleone P, Maj M. Dysfunctions of leptin, ghrelin, BDNF and endocannabinoids in eating disorders: Beyond the homeostatic control of food intake. Psychoneuroendocrinology. 2013;38(3):312–30. 10.1016/j.psyneuen.2012.10.021 23313276

[20] Wild B, Wesche D, Schultz J-H, Stroe-Kunold E, Herzog W, Rudofsky G, et al Trajectories of the cortisol awakening responses during weight gain in anorexia nervosa patients with severe and less severe symptoms. International Journal of Psychophysiology. 2014;94(3):272–7. 10.1016/j.ijpsycho.2014.09.010 25286448

[21] Brockmeyer T, Holtforth MG, Bents H, Kämmerer A, Herzog W, Friederich H-C. Starvation and emotion regulation in anorexia nervosa. Comprehensive psychiatry. 2012;53(5):496–501. 10.1016/j.comppsych.2011.09.003 22036318

[22] Rybakowski F, Slopien A, Tyszkiewicz-Nwafor M. Inverse relationship between leptin increase and improvement in depressive symptoms in anorexia nervosa. Neuroendocrinology Letters. 2014;35(1):64–7. 24625915

[23] Lu X-Y. The leptin hypothesis of depression: a potential link between mood disorders and obesity? Current Opinion in Pharmacology. 2007;7(6):648–52. 10.1016/j.coph.2007.10.010 18032111 PMC2677994

[24] Hebebrand J, Albayrak Ö. Leptin treatment of patients with anorexia nervosa? The urgent need for initiation of clinical studies. European Child and Adolescent Psychiatry. 2012;21(2):63–6. 10.1007/s00787-012-0243-3 22290117

[25] Wild B, Stadnitski T, Wesche D, Stroe-Kunold E, Schultz J-H, Rudofsky G, et al Temporal relationships between awakening cortisol and psychosocial variables in inpatients with anorexia nervosa–a time series approach. Int J Psychophysiol. 2016;102:25–32. 10.1016/j.ijpsycho.2016.03.002 26948136

[26] Stroe-Kunold E, Wesche D, Friederich H-C, Herzog W, Zastrow A, Wild B. Temporal Relationships of Emotional Avoidance in a Patient with Anorexia Nervosa—A Time Series Analysis. The International Journal of Psychiatry in Medicine. 2012;44(1):53–62. 23356093 10.2190/PM.44.1.d

[27] Löwe B, Wahl I, Rose M, Spitzer C, Glaesmer H, Wingenfeld K, et al A 4-item measure of depression and anxiety: Validation and standardization of the Patient Health Questionnaire-4 (PHQ-4) in the general population. Journal of Affective Disorders. 2010;122(1–2):86–95. 10.1016/j.jad.2009.06.019 19616305

[28] Serpell L, Teasdale JD, Troop NA, Treasure J. The development of the P-CAN, a measure to operationalize the pros and cons of anorexia nervosa. International Journal of Eating Disorders. 2004;36(4):416–33. 10.1002/eat.20040 15558651

[29] Fairburn CG, Beglin SJ. Assessment of eating disorders: Interview or self-report questionnaire? International Journal of Eating Disorders. 1994;16(4):363–70. 7866415

[30] Hilbert A, Tuschen-Caffier B, Karwautz A, Niederhofer H, Munsch S. Eating Disorder Examination-Questionnaire. Diagnostica. 2007;53(3):144–54.

[31] Singer JD. Using SAS PROC MIXED to Fit Multilevel Models, Hierarchical Models, and Individual Growth Models. Journal of Educational and Behavioral Statistics. 1998;23(4):323–55.

[32] Herpertz S, Albers N, Wagner R, Pelz B, Kopp W, Mann K, et al Longitudinal changes of circadian leptin, insulin and cortisol plasma levels and their correlation during refeeding in patients with anorexia nervosa. European Journal of Endocrinology. 2000;142(4):373–9. 10754479 10.1530/eje.0.1420373

[33] Hebebrand J, Muller TD, Holtkamp K, Herpertz-Dahlmann B. The role of leptin in anorexia nervosa: clinical implications. Mol Psychiatry. 2007;12(1):23–35. 10.1038/sj.mp.4001909 17060920

[34] Holtkamp K, Hebebrand J, Mika C, Heer M, Heussen N, Herpertz-Dahlmann B. High serum leptin levels subsequent to weight gain predict renewed weight loss in patients with anorexia nervosa. Psychoneuroendocrinology. 2004;29(6):791–7. 10.1016/S0306-4530(03)00143-4 15110928

[35] Janas-Kozik M, Stachowicz M, Krupka-Matuszczyk I, Szymszal J, Krysta K, Janas A, et al Plasma levels of leptin and orexin A in the restrictive type of anorexia nervosa. Regulatory Peptides. 2011;168(1–3):5–9. 10.1016/j.regpep.2011.02.005 21338627

[36] Lob S, Pickel J, Bidlingmaier M, Schaaf L, Backmund H, Gerlinghoff M, et al Serum leptin monitoring in anorectic patients during refeeding therapy. Experimental and Clinical Endocrinology and Diabetes 2003;111(5):278–82. 10.1055/s-2003-41286 12951634

[37] Seitz J, Bühren K, Biemann R, Timmesfeld N, Dempfle A, Winter SM, et al Leptin levels in patients with anorexia nervosa following day/inpatient treatment do not predict weight 1 year post-referral. Eur Child Adolesc Psychiatry. 2016. Epub 2016/02/06. 10.1007/s00787-016-0819-4 26847072

[38] Lu X-Y, Kim CS, Frazer A, Zhang W. Leptin: A potential novel antidepressant. Proceedings of the National Academy of Sciences. 2006;103(5):1593–8. 10.1073/pnas.0508901103 PMC1360555 16423896

[39] Liu J, Guo M, Lu X-Y. Leptin/LepRb in the Ventral Tegmental Area Mediates Anxiety-Related Behaviors. International Journal of Neuropsychopharmacology. 2015;10 2015. 10.1093/ijnp/pyv115 PMC4772826 26438799

[40] Lawson EA, Miller KK, Blum JI, Meenaghan E, Misra M, Eddy KT, et al Leptin levels are associated with decreased depressive symptoms in women across the weight spectrum, independent of body fat. Clinical Endocrinology. 2012;76(4):520–5. 10.1111/j.1365-2265.2011.04182.x 21781144 PMC3296868

[41] Stieg MR, Sievers C, Farr O, Stalla GK, Mantzoros CS. Leptin: A hormone linking activation of neuroendocrine axes with neuropathology. Psychoneuroendocrinology. 2015;51:47–57. Epub 2014/10/08. 10.1016/j.psyneuen.2014.09.004 25290346

[42] Ruscica M, Macchi C, Gandini S, Morlotti B, Erzegovesi S, Bellodi L, et al Free and bound plasma leptin in anorexia nervosa patients during a refeeding program. Endocrine. 2015:1–4. 10.1007/s12020-015-0598-6 25863491

[43] Treasure J, Corfield F, Cardi V. A Three-phase Model of the Social Emotional Functioning in Eating Disorders. European Eating Disorders Review. 2012;20(6):431–8. 10.1002/erv.2181 22539368

[44] Oldershaw A, Hambrook D, Stahl D, Tchanturia K, Treasure J, Schmidt U. The socio-emotional processing stream in anorexia nervosa. Neuroscience and Biobehavioral Reviews. 2011;35(3):970–88. 10.1016/j.neubiorev.2010.11.001 21070808

[45] Hatch A, Madden S, Kohn M, Clarke S, Touyz S, Williams L. Anorexia nervosa: towards an integrative neuroscience model. European Eating Disorders Review. 2010;18(3):165 10.1002/erv.974 20443202

[46] Haynos AF, Fruzzetti AE. Anorexia Nervosa as a Disorder of Emotion Dysregulation: Evidence and Treatment Implications. Clinical Psychology: Science and Practice. 2011;18(3):183–202.

[47] Burguera B, Couce ME, Long J, Lamsam J, Laakso K, Jensen MD, et al The Long Form of the Leptin Receptor (OB-Rb) Is Widely Expressed in the Human Brain. Neuroendocrinology. 2000;71(3):187–95. 10729790 10.1159/000054536

[48] Adolphs R. Neural systems for recognizing emotion. Current Opinion in Neurobiology. 2002;12(2):169–77. 12015233 10.1016/s0959-4388(02)00301-x

[49] Paz‐Filho G, Wong ML, Licinio J. The procognitive effects of leptin in the brain and their clinical implications. International journal of clinical practice. 2010;64(13):1808–12. 10.1111/j.1742-1241.2010.02536.x 21070531 PMC2998704

